# Serum Expression of *β*-Catenin Is a Potential Detection Marker in Patients with Colorectal Cancer

**DOI:** 10.1155/2019/5070524

**Published:** 2019-10-24

**Authors:** Shue Li, Mao Huang, Qiao Liu, Ding Wang, Rui Wu, Xiuyu Zhang, Weixian Chen, Liang Duan

**Affiliations:** ^1^Department of Laboratory Medicine, The Second Affiliated Hospital of Chongqing Medical University, Chongqing 400010, China; ^2^Department of Academic Research, The Second Affiliated Hospital of Chongqing Medical University, Chongqing 400010, China; ^3^Key Laboratory of Clinical Laboratory Diagnostics, Ministry of Education, College of Laboratory Medicine, Chongqing Medical University, Chongqing 400010, China; ^4^Department of Laboratory Medicine, The First Affiliated Hospital of Chongqing Medical University, Chongqing 400010, China

## Abstract

**Object:**

To investigate the correlation between the level of serum *β*-catenin and the disease progression of colorectal polyp (CRP) and colorectal cancer (CRC) and find its potential diagnostic value.

**Methods:**

A total of 327 clinical serum samples and their electronic medical records were collected. Detecting by enzyme-linked immunosorbent assay (ELISA), the correlations of serum *β*-catenin with tumor marker carcinoembryonic antigen (CEA) and CRC clinicopathological parameters and the receiver operating characteristic (ROC) curve were analyzed.

**Results:**

Serum *β*-catenin levels in the CRP and CRC patients were significantly higher than those in the healthy control (HC) group (*P* < 0.05 and *P* < 0.001). Compared with CRP, serum *β*-catenin level in CRC was also increased (*P* < 0.05). However, there was no significant difference in gender, age, location, tumor size, Dukes staging, or metastasis (*P* > 0.05) between serum *β*-catenin and clinical parameters of CRC. There was no correlation between serum *β*-catenin levels and CEA in CRC patients (*P* = 0.14). ROC curve analysis showed that serum *β*-catenin possessed the maximum diagnostic efficiency in CRP (AUC = 0.73, *P* < 0.05) with 86.41% sensitivity and 51.56% specificity. *β*-Catenin combined with CEA had the highest diagnostic efficiency (AUC = 0.88, *P* < 0.05) with 81.88% sensitivity and 73.44% specificity. With CRC patients from CRP patients, ROC analysis of the combining detection (AUC = 0.70, *P* < 0.05) had the 70% sensitivity and 84.5% specificity.

**Conclusion:**

The serum *β*-catenin levels are gradually increased in CRP and CRC, while there is no correlation between its levels and CRC disease process. Single serum *β*-catenin or combined CEA would be one of the potential candidate biomarkers for colorectal disease diagnosis.

## 1. Introduction

Colorectal cancer (CRC) is one of the most common malignant tumors in the digestive system [1]. Globally, it is third in morbidity and fourth in mortality [2]. It becomes a large burden in the world. CRC is a multistage process, which is that healthy colonic epithelium could slowly develop into polyps or adenomas and then into carcinomas over 20-40 years [3, 4]. Current methods for clinical diagnosis of colorectal cancer include stool tests, colonoscopy [5], and colonography (MRI and CT) [6, 7]. However, stool tests have their limitations in terms of sensitivity and specificity [8], while colonoscopy needs for the higher tolerance of patients and colonography needs for appropriate bowel preparation. Extensive efforts have been made in the past and are currently underway for the development of molecular markers in CRC. These molecular markers should be unique, noninvasive, safe, and affordable with high sensitivity and specificity [9]. To facilitate better clinical decision-making, many researchers work on finding out which biomarker can detect CRC at an early stage, like nucleic acids, lipid, proteins, and tumor cells [10–12]. But so far, little success has been achieved in this area, and searching for a noninvasive serum tumor marker with high sensitivity and specificity is still needed.

Wingless-related integration site (*Wnt*)/*β*-catenin signaling pathway is a conserved pathway mediating the tumorigenesis and embryogenesis [13], especially in the procession of CRC [14, 15]. *Wnt* signaling regulates signal transduction by controlling the levels of *β*-catenin through phosphorylation and proteasomal degradation [9]. The earliest and most prevalent genetic event in colorectal tumorigenesis is the mutation of adenomatous polyposis coli (APC) gene [16]. Inactivation of APC leads to the disassembling of the *β*-catenin destruction complexes (Axin, GSK3*β*, and APC) and then the accumulation of *β*-catenin in the cytoplasm and nucleus [17]. In the past, many efforts mainly focused on the accumulation of nucleus *β*-catenin in CRC cells, which initiates the transcription of many target genes that related to proliferation and invasion and then leads to tumorigenesis [18].

Recent studies have found that *β*-catenin can be detected in human serum, and its level is related to the development of hepatitis C-related liver cancer, hepatitis B-related diseases, type 2 diabetes, PTEN hamartoma tumor syndrome, and early-onset ulcerative colitis [19–23]. Given that there is a hyperactivated *Wnt*/*β*-catenin signal in CRC and a large amount of *β*-catenin aggregation in intracellular and nucleus, the level of serum *β*-catenin in CRC patients and related clinical significance are still unclear. Therefore, our research detected the level of serum *β*-catenin in CRP and CRC to analyze the potential role in disease diagnosis.

## 2. Materials and Method

### 2.1. Patients and Sample Collection

The serum samples (*n* = 327) were recruited from the Second affiliated hospital of Chongqing Medical University from January 2015 to March 2018. The samples were divided into three groups, CRC group (*n* = 160), CRP group (*n* = 103), and normal healthy subjects with no history of CRC or CRP disease group (*n* = 64). CRC group included 86 males and 74 females: 95 patients below the age of 60 years and 65 patients beyond 60 years old. According to the tumor size, 78 cases of the tumors were less than 3 cm and 82 cases were over 3 cm. According to tumor location, 83 cases were with colon tumors and 77 cases were with rectal tumors. According to the Dukes staging system, colorectal tumors were divided into stages A, B, C, and D. 81 cases were in A+B stage, and 79 cases were in C+D stage. There were 95 cases of nonmetastasis and 65 cases of lymphatic metastasis in CRC patients (Table 1). All the patients had no other tumors and severe organic diseases. All the volunteers, with informed consent, took 4 mL of blood through the peripheral vein on an empty stomach. The serum with non-anticoagulation treatment was sealed and numbered and then stored at -80°C for testing. Eight pairs of fresh CRC tissues and matching distal normal tissues and five CRP tissues were collected from patients who had undergone colorectal resection at the First Affiliated Hospital of the Chongqing Medical University. The patients received no chemotherapy, hormonal therapy, or radiotherapy before surgery, and written informed consent was received from all participants. The study was approved by the Institutional Ethics Committee for human studies at Chongqing Medical University, Chongqing, China (protocol number 2012-09). All the procedures were following the Declaration of Helsinki.

### 2.2. ELISA

The serum *β*-catenin level in all samples was measured by a commercially available enzyme-linked immunosorbent assay kit (ELISA) (CUSABIO, Wuhan, China) according to the manufacturer's instructions. The CEA values were measured by electrochemiluminescence immunoassay “ECLIA”.

### 2.3. Immunocytochemistry Staining (IHC)

This experiment was detected according to Wu et al.'s method [24], briefly described in the following: firstly, the tissues were formalin-fixed and then paraffin-embedded. The sections from the tissues were deparaffinized and dehydrated. They were boiled for 10 min in 0.01 M citrate buffer and incubated with 0.3% hydrogen peroxide (H_2_O_2_) in methanol for 15 min to block endogenous peroxidase. The sections were then incubated with the anti-*β*-catenin polyclonal antibody (1 : 300 dilution; sc-7199, Santa Cruz) overnight at 4°C, following incubation with secondary antibody tagged with the peroxidase enzyme (SP-9000, Zhongshan Golden Bridge, China) for 30 min at room temperature, and were visualized with 0.05% 3,3-diamino-benzidine tetrachloride (DAB) till the desired brown reaction product was obtained. The sections were finally counterstained with hematoxylin. Control sections were performed using phosphate-buffered solution (PBS) without a primary antibody. All the slides were observed under a Nikon E400 Light microscope, and representative images were taken.

### 2.4. Statistical Analyses

All the data were processed with SPASS 20.0 software. The difference between serum *β*-catenin and CEA level in CRP and CRC patients and HC was analyzed by the Kruskal-Wallis test. The relationship between serum *β*-catenin and clinical pathological parameters of CRC was analyzed by the Mann–Whitney test. The ROC curve analysis was conducted to evaluate the diagnostic efficacy of serum *β*-catenin, CEA, and “*β*-catenin+CEA” in samples. The correlation between serum *β*-catenin and CEA level was estimated by Spearman's rank correlation coefficient. The statistical differences are presented at probability levels of *P* < 0.05 (∗), *P* < 0.01 (∗∗), and *P* < 0.001 (∗∗∗), indicating that the difference was statistically significant.

## 3. Results

### 3.1. The Differential Expression Level Analysis of Serum *β*-Catenin in HC, CRP, and CRC Groups

**Figure 1:**
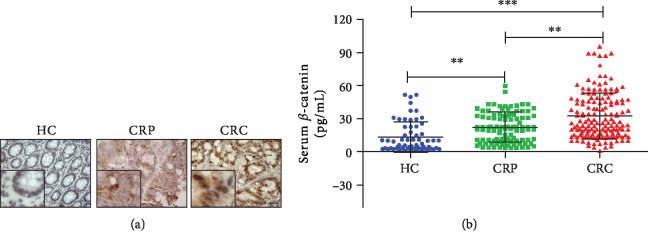
The differential expression level analysis of serum *β*-catenin in healthy control (HC), colorectal polyp (CRP), and colorectal cancer (CRC) group. (a) The IHC staining for *β*-catenin in representative samples. The blank scale bars = 100 *μ*m. (b) The serum levels of *β*-catenin in CRP and CRC patients and HC. ^∗∗∗^*P* < 0.001, CRC vs. HC; ^∗∗^*P* < 0.01, CRC vs. CRP or CRP vs. HC.

We performed IHC staining to detect the expression of *β*-catenin in sections of eight pairs of samples (CRC and matching distal normal tissues) and five CRP samples. Expression levels of *β*-catenin were significantly higher from either CRC or CRP tissues than from HCs. Of note, its expression was higher in CRC tissues than that in CRP tissues (Figure 1(a)). Further, the serum *β*-catenin level in the CRP group (21.81 ± 13.37, pg/mL) and CRC group (32.13 ± 20.53, pg/mL) was higher than that in the HC group (12.85 ± 13.78, pg/mL). The difference was statistically significant (*P* < 0.05 and *P* < 0.001, Figure 1(b)). Simultaneously, the serum *β*-catenin level of CRC group (32.13 ± 20.53, pg/mL) was higher than that of the CRP group (21.81 ± 13.37, pg/mL) (*P* < 0.05, Figure 1(b)). These results showed that the level of *β*-catenin gradually increases during the processing of the development from CRP to CRC.

### 3.2. The Correlation between the Expression Level of Serum *β*-Catenin and Clinicopathological Factors of CRC

Given the significant increase in the level of serum *β*-catenin for CRC patients, does it relate to the process of CRC development? According to the different clinicopathological factors of CRC, we classified 160 CRC patients. First, the relationship between serum *β*-catenin level and gender, age, and the location was analyzed. We found that the serum *β*-catenin level in the male group was 33.52 ± 21.28 pg/mL and in the female group was 30.1 ± 19.35 pg/mL. This difference was little statistically significant (*P* = 0.34). The serum *β*-catenin level in the group younger than 60 was 30.22 ± 18.51 pg/mL and in the group older than 60 was 34.35 ± 22.57 pg/mL. This difference was not statistically significant (*P* = 0.34). The serum *β*-catenin level was 31.45 ± 19.9 pg/mL in the colon tumor group and 32.86 ± 21.25 pg/mL in the rectal tumor group. The difference was little statistically significant (*P* = 0.76). Also, we analyzed the relationship with essential parameters for the process of CRC disease, like tumor size, Dukes stages, and lymph node metastasis. The serum *β*-catenin level is 31.92 ± 21.7 pg/mL in the tumor group (size < 3 cm), and 32.33 ± 19.47 pg/mL in the tumor group (size ≥ 3 cm), with no significant difference (*P* = 0.57). We also found that the serum *β*-catenin level in Dukes stages (A+B) is 32.67 ± 20.36 pg/mL, and 31.58 ± 20.82 pg/mL in Dukes stages (C+D), with no significant difference (*P* = 0.7). The serum *β*-catenin level in the nontransfer (Absent) group is 31.79 ± 20.23 pg/mL, and 32.23 ± 21 pg/mL in the transfer (Present) group, with no significant difference (*P* = 0.86). These results were shown in Table 2.

### 3.3. The Correlation between the Expression of CEA and Serum *β*-Catenin Level in Colorectal Cancer

**Figure 2:**
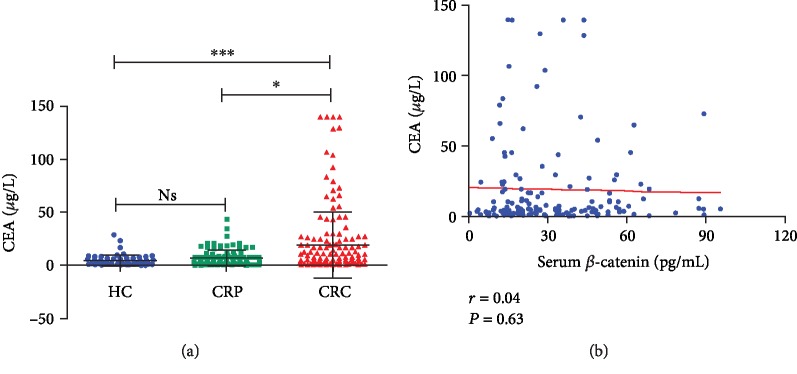
The levels of CEA in CRP and CRC patients and the correlation analysis between CEA and *β*-catenin. (a) The CAE level in serum for healthy control (HC), CRP patients, and CRC patients. ^∗∗∗^*P* < 0.001, CRC vs. HC; ^∗^*P* < 0.05, CRC vs. CRP; Ns (not significant), CRP vs. HC. (b) The correlation analysis between CEA and *β*-catenin, *r* = 0.04, *P* = 0.63.

As a relatively better diagnostic marker for CRC, does CEA have any correlation with serum *β*-catenin? We used the electrochemiluminescence immunoassay “ECLIA” technique to detect the CEA level in the collected samples. We found that the CEA level in the CRC group (14.27 ± 26*μ*g/L) is significantly higher than that in the CRP group (7.13 ± 7.52*μ*g/L) and the HC group (4.34 ± 4.98*μ*g/L) (*P* < 0.05, Figure 2(a)). The CRP group showed no level of statistical difference (*P* > 0.05, Figure 2(a)) with the HC group. No correlation was shown between serum *β*-catenin level and serum CEV level in CRC patients (*r* = 0.04, *P* > 0.05) by Spearman's rank correlation analysis (Figure 2(b)).

### 3.4. The Clinical Diagnosis Value of Serum *β*-Catenin Level in CRP and CRC

**Figure 3:**
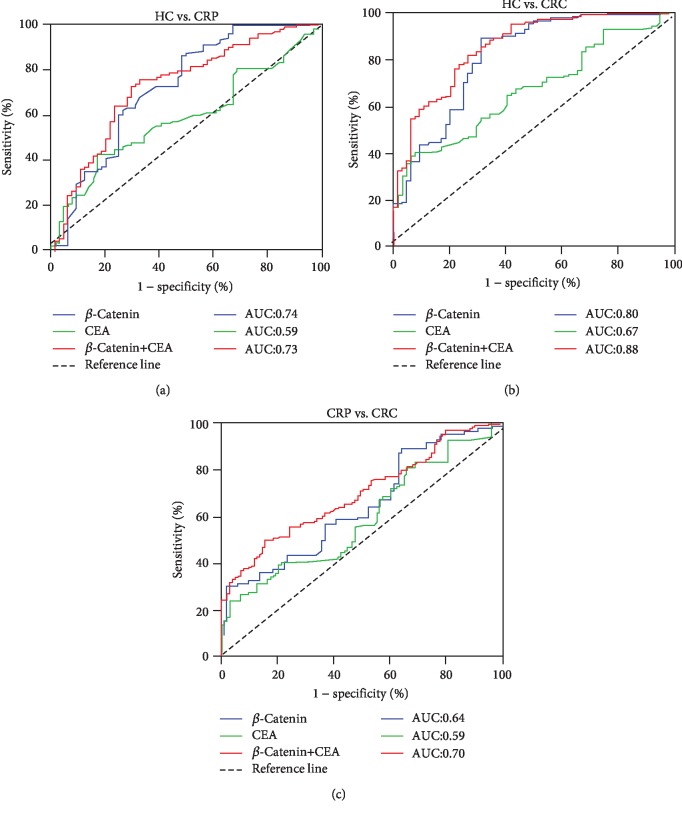
The diagnostic efficacy analysis of serum *β*-catenin and/or CEA in CRP and CRC. (a) Analysis of ROC curve in patients with CRP diagnosed by serum *β*-catenin and CEA alone or in combination. (b) Analysis of ROC curve in patients with CRC diagnosed by serum *β*-catenin and CEA alone or in combination. (c) The ROC curve analysis of serum *β*-catenin and CEA in patients with CRC diagnosed by CRP alone or in combination.

As the level of serum *β*-catenin increases gradually during the processing of the development from CRP to CRC, does it have any diagnostic value in the process of the disease and would be a better diagnostic value when combining with CEA value which is the existing marker? We divided these indicators into three groups in this section, followed as a *β*-catenin group, CEA group, and *β*-catenin+CEA group. The diagnostic efficacy of these groups was analyzed using the ROC curves. When CRP occurs, the AUC in serum *β*-catenin group, CEA group, and *β*-catenin+CEA group were 0.74, 0.59, and 0.73, respectively. It suggested that serum *β*-catenin has the highest diagnostic efficacy. The sensitivity of serum *β*-catenin is 86.41%, and the specificity is 51.56% (Figure 3(a)). When CRC occurs, the AUC in serum *β*-catenin group, CEA group, and *β*-catenin+CEA group were 0.80, 0.67, and 0.88, respectively. It suggested that *β*-catenin combined with CEA has the highest diagnostic efficacy. The sensitivity of combined *β*-catenin and CEA was 81.88%, and the specificity was 73.44% (Figure 3(b)). When CRP developed to CRP, the AUC in serum *β*-catenin group, CEA group, and *β*-catenin+CEA group were 0.64, 0.59, and 0.70, respectively. It suggested that the diagnostic efficacy of combined *β*-catenin and CEA was comparatively high. The sensitivity of *β*-catenin combined with CEA is 70%, and the specificity is 84.5% (Figure 3(c)). These results suggest that the combination of serum *β*-catenin and/or CEA level has better diagnostic potential in the process of CRP, even in the process of CRC disease.

## 4. Discussion

Colorectal cancer is one of the most common malignant tumors that threaten human health [25]. Although the improved living standards and the changes of lifestyles and dietary patterns, the incidence of colorectal cancer is still increasing [21]. Compared with fecal excretion examination, colorectal examination, CT colon imaging examination, or MRI examination methods, the serum tumor biomarkers in the diagnosis and treatment of colorectal cancer are convenient material extraction, small trauma, high reproducibility, and lower cost of detection. Therefore, serum cancer biomarkers have become a hot spot for tumor diagnosis and detection. Single serum *β*-catenin or combined CEA has a better diagnostic value of CRP and CRC. It would be one of the potential candidate biomarkers for colorectal disease diagnosis.

The sample in the peripheral blood includes circulating tumor cells (CTC); cell-free DNA (cfDNA), which includes circulating tumor DNA (ctDNA); circulating cell-free RNA (cfRNA), which includes not only some small RNAs but also mRNAs; circulating extracellular vesicles (EVs); proteins; and metabolites [26]. ctDNA could accumulate in the blood stream due to the release of ctDNA from dying tumor cells during necrosis or apoptosis. Under normal conditions, the innate immune system timely clears the cell breakdown products, thus staying in low concentration in the blood stream. In tumors, rapid cell turnover results in accumulation of cell products, including ctDNA which often is not accessible to phagocytes [26]. Those methods to look for evidence of cancer circulating in the blood could be defined as liquid biopsy. The use of ctDNA and CTCs as an alternative screening tool for CRC is under investigation. A study found that the ctDNA was positive in 73% in localized CRC and levels of ctDNA correlated with disease stage, thus suggesting that ctDNA can be used both in localized disease and metastatic CRC [27]. Three cases of DNA-gene mutations (CDKN2A, TP53, and KRAS) were observed 4 years prior to CRC formation [28]. Especially, KRAS mutant fragments were detected in the blood of patients with KRAS-mutant colorectal tumors, with high specificity (99.2%) and sensitivity (87.2%) [29]. During a median follow-up time of 9.2 person-years, the DNA aneuploidy of 53 patients (total 245 patients) was found which was estimated by Kaplan-Meier curves [30]. Thus, DNA aneuploidy also might predict the CRC formation. However, several decades devoted to elevate ctDNA levels in the circulation of patients with cancer. The quantity of ctDNA and CTCs detected has been shown to correlate with tumor burden and enables assessment of heterogeneity. That means plasma often contains low levels of ctDNA in early or localized disease, which becomes a big challenge for the use of liquid biopsy as a screening tool.

*β*-Catenin is a crucial component of the Wingless-related integration site (*Wnt*) signaling pathway. High expression of *β*-catenin is considered a sign of aberrant *β*-catenin signaling pathway activation and is thought to promote tumor progression [31], especially CRC progression [32]. The start signals for the activation of the *Wnt* signaling pathway were *Wnt* proteins, which bind to the frizzled (Fz) receptors and coreceptor LRP on the cell membrane [33, 34]. The absence of *Wnt* ligands caused the phosphorylation of *β*-catenin. The phosphorylated *β*-catenin fails to bind and disperse the components of the degraded complex. Thus, the *β*-catenin cannot be degraded, resulting in the accumulation of *β*-catenin in cells [13, 35]. This theory could support a new and effective therapy of colorectal cancer, that is, using a certain molecular substance which could attenuate the *Wnt* signaling pathway to inhibit cell growth and tumor growth. Studies on *β*-catenin mainly involved in the accumulation in the cell and the transcription of the target gene into the nucleus, leading to tumorigenesis [18, 36–38]. Recently, a small number of cases have confirmed that *β*-catenin can be detected in serum and might have a certain relationship with the course of hepatitis C, hepatitis B, type 2 diabetes, PTEN hamartoma tumor syndrome, and early-onset ulcerative colitis [19–23]. These findings suggested that *β*-catenin might be used as a marker for serum disease-associated markers. Many studies focused on the initiation of target genes in the cell by *β*-catenin into the nucleus, but neglected that it may itself be a cytokine that can be secreted outside the extracellular. As a multifunctional protein molecule, *β*-catenin can also be combined with E-cadherin to mediate cell adhesion. Under normal circumstances, *β*-catenin is abundantly located on the cell membrane, while in the stages of tumor disease, EMT phenomenon is often accompanied by a decrease in E-cadherin [39]. Thus, it is suspected that the reduction in E-cadherin may result in the inability of *β*-catenin to bind and release into the extracellular space. However, this hypothesis needs to be further confirmed.

This study showed that the serum *β*-catenin level in CRP and CRC patients was significantly higher than that in healthy controls. The serum *β*-catenin level of the CRC patients was also significantly increased compared with that of the CRP group. These results indicated that the serum *β*-catenin level tends to increase gradually in the process of colorectal polyps or colorectal cancer. To explore the relationship between serum *β*-catenin and CRC disease progression, we grouped these cases according to the factors, e.g., gender, age, tumor size, tumor location, metastasis, and Dukes staging. We found that the level of serum *β*-catenin in CRC patients was not associated with these pathological parameters. Therefore, when the serum *β*-catenin level of CRC patients is elevated, we can only reasonably suspect the possibility of early development for CRC, but could not estimate the stage of the disease.

Furthermore, carcinoembryonic antigen (CEA) is a common cancer biomarker. It is a high molecular weight glycoprotein found in embryonic tissues and colorectal malignancies. High CEA levels are associated with tumorigenesis. Many tumorigeneses in human were found in the increase of serum CEA level, and it is better for efficacy judgment, disease development, detection, and pretreatment for colorectal cancer, breast cancer, and lung cancer [40–43]. Investigators have found that ctDNA in combination with CEA is a potentially useful tool for the diagnosis of early-stage colorectal cancer [27]. The increase of CEA level in primary CRC patients has been confirmed [44]. However, CEA is not specific for CRC and can be elevated in pancreaticobiliary disease, inflammatory bowel disease (IBD), and other malignancies [45, 46]. Thus, we also conducted a correlation study between the serum *β*-catenin level and the CEA level. The Spearman rank correlation coefficient analysis showed no correlation between the two factors. This phenomenon might be due to the limitations of CEA, which had lower specificity and sensitivity. It is difficult to capture the changes in the early stages of the tumor, and *β*-catenin may change at an earlier stage.

Finally, we used the ROC curves to assess the diagnostic value of serum *β*-catenin in the process of CRP to CRC disease. The results showed that serum *β*-catenin had the highest diagnostic efficacy in the diagnosis of CRP (AUC = 0.74). In the evaluation of CRC, *β*-catenin combined with CEA had the highest diagnostic efficacy (AUC = 0.88). Also, we evaluated the detection efficiency of CRC in CRP, which determined by *β*-catenin combined with CEA. The AUC was 0.70. These results demonstrated that serum *β*-catenin level could detect changes in early colorectal disease and combined with CEA can improve the diagnostic accuracy of CRC.

## 5. Conclusions

In sum, CRP and CRC have a significant increase in the level of serum *β*-catenin. It has little correlation with the disease progression of CRC. While combining the value of CEA, it may improve the accuracy of the early diagnosis of colorectal diseases. The diagnostic performance of CRC is fast and straightforward. Therefore, these studies not only have a role in screening and early detection of disease but are also important for prognosis and to predict response to therapy in CRC, reducing its morbidity and mortality.

## Figures and Tables

**Table 1:** The clinicopathological parameters of collected serum samples.

Parameters	CRC (*n* = 160), *n* (%)	CRP (*n* = 103), *n* (%)	HC (*n* = 64), *n* (%)
Gender			
Male (*n*, %)	86 (53.7%)	53 (51.5%)	39 (61%)
Female (*n*, %)	74 (46.3%)	50 (48.5%)	25 (39%)
Age			
<60 (*n*, %)	95 (59.4%)	69 (70%)	24 (37.5%)
≥60 (*n*, %)	65 (40.6%)	34 (30%)	40 (62.5%)
Location			
Colon (*n*, %)	83 (51.9%)	43 (42%)	NA
Rectum (*n*, %)	77 (48.1%)	60 (58%)	
Tumor size			
<3 cm (*n*, %)	78 (48.8%)	NA	NA
≥3 cm (*n*, %)	82 (51.2%)	NA	NA
Dukes staging			
A/B (*n*, %)	81 (50.6%)	NA	NA
C/D (*n*, %)	79 (49.4%)	NA	NA
Metastasis			
Absent (*n*, %)	95 (59.4%)	NA	NA
Present (*n*, %)	65 (40.6%)	NA	NA

**Table 2:** Correlations of serum *β*-catenin level and different clinicopathological parameters of CRC.

Parameters	CRC (*n* = 160), *n* (%)	*β*-Catenin (*x* ± SD (pg/mL))	*P* value
Gender			
Male (*n*, %)	86 (53.7%)	33.52 ± 21.28	*P* = 0.34
Female (*n*, %)	74 (46.3%)	30.1 ± 19.35	
Age			
<60 (*n*, %)	95 (59.4%)	30.22 ± 18.51	*P* = 0.34
≥60 (*n*, %)	65 (40.6%)	34.35 ± 22.57	
Location			
Colon (*n*, %)	83 (51.9%)	31.45 ± 19.9	*P* = 0.76
Rectum (*n*, %)	77 (48.1%)	32.86 ± 21.25	
Tumor size			
<3 cm (*n*, %)	78 (48.8%)	31.92 ± 21.7	*P* = 0.57
≥3 cm (*n*, %)	82 (51.2%)	32.33 ± 19.47	
Dukes staging			
A/B (*n*, %)	81 (50.6%)	32.67 ± 20.36	*P* = 0.7
C/D (*n*, %)	79 (49.4%)	31.58 ± 20.82	
Metastasis			
Absent (*n*, %)	95 (59.4%)	31.79 ± 20.23	*P* = 0.86
Present (*n*, %)	65 (40.6%)	32.23 ± 21	

## Data Availability

The data used to support the findings of this study are included within the article.

## References

[1] Qi L., Zhang W., Cheng Z., Tang N., Ding Y. (2017). Study on molecular mechanism of ANOS1 promoting development of colorectal cancer. *PLoS One*.

[2] Amado N., Predes D., Moreno M., Carvalho I., Mendes F., Abreu J. (2014). Flavonoids and Wnt/*β*-catenin signaling: potential role in colorectal cancer therapies. *International Journal of Molecular Sciences*.

[3] Michor F., Iwasa Y., Lengauer C., Nowak M. A. (2005). Dynamics of colorectal cancer. *Seminars in Cancer Biology*.

[4] Fearon E. R., Vogelstein B. (1990). A genetic model for colorectal tumorigenesis. *Cell*.

[5] Quintero E., Castells A., Bujanda L. (2012). Colonoscopy versus fecal immunochemical testing in colorectal-cancer screening. *The New England Journal of Medicine*.

[6] Barish M. A., Rocha T. C. (2005). Multislice CT colonography: current status and limitations. *Radiologic Clinics of North America*.

[7] van der Paardt M. P., Stoker J. (2018). Current status of magnetic resonance colonography for screening and diagnosis of colorectal cancer. *Radiologic Clinics of North America*.

[8] De Rosa M., Pace U., Rega D. (2015). Genetics, diagnosis and management of colorectal cancer (review). *Oncology Reports*.

[9] Shukla K. K., Sharma P., Misra S. (2019). *Molecular Diagnostics in Cancer Patients*.

[10] Yan G., Li L., Zhu B., Li Y. (2016). Lipidome in colorectal cancer. *Oncotarget*.

[11] Xue M., Lai S. C., Xu Z. P., Wang L. J. (2015). Noninvasive DNA methylation biomarkers in colorectal cancer: a systematic review. *Journal of Digestive Diseases*.

[12] Das V., Kalita J., Pal M. (2017). Predictive and prognostic biomarkers in colorectal cancer: a systematic review of recent advances and challenges. *Biomedicine and Pharmacotherapy*.

[13] Pai S. G., Carneiro B. A., Mota J. M. (2017). Wnt/beta-catenin pathway: modulating anticancer immune response. *Journal of Hematology & Oncology*.

[14] Sebio A., Kahn M., Lenz H.-J. (2014). The potential of targeting Wnt/*β*-catenin in colon cancer. *Expert Opinion on Therapeutic Targets*.

[15] Huang G., Zhu H., Shi Y., Wu W., Cai H., Chen X. (2015). cir-ITCH plays an inhibitory role in colorectal cancer by regulating the Wnt/*β*-catenin pathway. *PLoS One*.

[16] Kinzler K. W., Nilbert M. C., Vogelstein B. (1991). Identification of a gene located at chromosome 5q21 that is mutated in colorectal cancers. *Science*.

[17] Barker N., Clevers H. (2000). Catenins, Wnt signaling and cancer. *BioEssays*.

[18] Lugli A., Zlobec I., Minoo P. (2007). Prognostic significance of the Wnt signalling pathway molecules APC, *β*-catenin and E-cadherin in colorectal cancer—a tissue microarray-based analysis. *Histopathology*.

[19] Zekri A. R., Zekri A.-R. N., Bahnassy A. A. (2011). Serum levels of *β*-catenin as a potential marker for genotype 4/hepatitis C-associated hepatocellular carcinoma. *Oncology Reports*.

[20] Duan L., Yang Q., Yang J. (2018). Identification of serum *β*-catenin as a biomarker in patients with HBV-related liver diseases. *Journal of Translational Medicine*.

[21] Gaudio A., Privitera F., Battaglia K. (2012). Sclerostin levels associated with inhibition of the Wnt/*β*-catenin signaling and reduced bone turnover in type 2 diabetes mellitus. *The Journal of Clinical Endocrinology and Metabolism*.

[22] Galatola M., Miele E., Strisciuglio C. (2013). Synergistic effect of interleukin-10-receptor variants in a case of early-onset ulcerative colitis. *World Journal of Gastroenterology*.

[23] Galatola M., Paparo L., Duraturo F. (2012). Beta catenin and cytokine pathway dysregulation in patients with manifestations of the "PTEN hamartoma tumor syndrome". *BMC Medical Genetics*.

[24] Wu R., Zhang Y., Xiang Y. (2018). Association between serum S100A9 levels and liver necroinflammation in chronic hepatitis B. *Journal of Translational Medicine*.

[25] Myung D.-S., Kweon S.-S., Lee J. (2017). Clinicopathological features of laterally spreading colorectal tumors and their association with advanced histology and invasiveness: an experience from Honam province of South Korea: a honam association for the study of intestinal diseases (HASID). *PLoS One*.

[26] Heitzer E., Haque I. S., Roberts C. E. S., Speicher M. R. (2019). Current and future perspectives of liquid biopsies in genomics-driven oncology. *Nature Reviews Genetics*.

[27] Umetani N., Kim J., Hiramatsu S. (2009). Increased integrity of free circulating DNA in sera of patients with colorectal or periampullary cancer: direct quantitative PCR for ALU repeats. *Clinical Chemistry*.

[28] Galandiuk S., Rodriguez–Justo M., Jeffery R. (2012). Field cancerization in the intestinal epithelium of patients with Crohn’s ileocolitis. *Gastroenterology*.

[29] Siravegna G., Marsoni S., Siena S., Bardelli A. (2017). Integrating liquid biopsies into the management of cancer. *Nature Reviews Clinical Oncology*.

[30] Söderlund S., Tribukait B., Öst Å. (2011). Colitis-associated DNA aneuploidy and dysplasia in Crohn’s disease and risk of colorectal cancer. *Inflammatory Bowel Diseases*.

[31] Lee S. B., Gong Y.-D., Park Y. I., Dong M. S. (2013). 2,3,6-Trisubstituted quinoxaline derivative, a small molecule inhibitor of the Wnt/beta-catenin signaling pathway, suppresses cell proliferation and enhances radiosensitivity in A549/Wnt2 cells. *Biochemical and Biophysical Research Communications*.

[32] Yoshida N., Kinugasa T., Ohshima K. (2015). Analysis of Wnt and *β*-catenin expression in advanced colorectal cancer. *Anticancer Research*.

[33] Nishioka M., Ueno K., Hazama S. (2013). Possible involvement of Wnt11 in colorectal cancer progression. *Molecular Carcinogenesis*.

[34] Varelas X., Miller B. W., Sopko R. (2010). The hippo pathway regulates Wnt/*β*-catenin signaling. *Developmental Cell*.

[35] Rahmani F., Avan A., Hashemy S. I., Hassanian S. M. (2018). Role of Wnt/*β*-catenin signaling regulatory microRNAs in the pathogenesis of colorectal cancer. *Journal of Cellular Physiology*.

[36] Kim C. J., Cho Y. G., Park Y. G. (2005). Pin1 overexpression in colorectal cancer and its correlation with aberrant *β*-catenin expression. *World Journal of Gastroenterology*.

[37] Shashar M., Siwak J., Tapan U. (2016). c-Cbl mediates the degradation of tumorigenic nuclear *β*-catenin contributing to the heterogeneity in Wnt activity in colorectal tumors. *Oncotarget*.

[38] Ghosh M., Sakhuja P., Singh S., Agarwal A. K. (2013). p53 and beta-catenin expression in gallbladder tissues and correlation with tumor progression in gallbladder cancer. *Saudi Journal of Gastroenterology*.

[39] Kobayashi M., Honma T., Matsuda Y. (2000). Nuclear translocation of beta-catenin in colorectal cancer. *British Journal of Cancer*.

[40] Huber A. H., Weis W. I. (2001). The structure of the *β*-catenin/E-cadherin complex and the molecular basis of diverse ligand recognition by *β*-catenin. *Cell*.

[41] Dong D., Jia L., Zhang L. (2018). Periostin and CA242 as potential diagnostic serum biomarkers complementing CA19.9 in detecting pancreatic cancer. *Cancer Science*.

[42] Wei P. L., Lee L. T., Tseng L. M., Huang K. W. (2018). Validation of assaying carcinoembryonic antigen in human serum by using immunomagnetic reduction. *Scientific Reports*.

[43] Rizeq B., Zakaria Z., Ouhtit A. (2018). Towards understanding the mechanisms of actions of carcinoembryonic antigen-related cell adhesion molecule 6 in cancer progression. *Cancer Science*.

[44] He M., Huang Z., Yan X. (2013). Label-free detection of hepatocellular carcinoma markers based on photoluminescence of antibody-conjugated ZnO arrays. *Journal of Biomedical Nanotechnology*.

[45] Locker G. Y., Hamilton S., Harris J. (2006). ASCO 2006 update of recommendations for the use of tumor markers in gastrointestinal cancer. *Journal of Clinical Oncology*.

[46] Hundt S., Haug U., Brenner H. (2007). Blood markers for early detection of colorectal cancer: a systematic review. *Cancer Epidemiology Biomarkers & Prevention*.

